# Short coiled‐coil proteins from plants and metazoans – the ‘jacks of all trades’

**DOI:** 10.1111/febs.70159

**Published:** 2025-06-22

**Authors:** Agnieszka Sirko, Jarosław Poznański, Marzena Sieńko

**Affiliations:** ^1^ Institute of Biochemistry and Biophysics Polish Academy of Sciences Warsaw Poland

**Keywords:** autophagy, cellular trafficking, LSU, response to low sulfur, SCOC

## Abstract

The molecular functions of short coiled‐coil proteins remain poorly characterized. These proteins typically act as facilitators rather than essential components of metabolic processes, contributing to cellular homeostasis, and are aptly described as ‘jacks of all trades but masters of none’. They are found across diverse groups of organisms, including both plants and animals. LSU (RESPONSE TO LOW SULFUR) are plant proteins induced under sulfur deficiency and other environmental stresses. They participate in metabolic pathways, including sulfate assimilation, and manage oxidative stress by stabilizing and protecting antioxidative enzymes. In metazoans, SCOC (SHORT COILED‐COIL) proteins regulate autophagy initiation by recruiting proteins essential for forming autophagosomes—key vesicles involved in cellular degradation. SCOC proteins also interact with factors critical for maintaining membrane dynamics and intracellular transport. Despite some functional similarities, the roles of these proteins have diverged significantly between plants and animals, reflecting organism‐specific adaptations shaped by evolutionary pressures. This divergence underscores their adaptive versatility and highlights their potential as promising targets for future biological research.

## Graphical abstract

Short coiled‐coil proteins from plants and metazoans—true ‘jacks of all trades’—play supporting but crucial roles in cellular stress response, metabolism, autophagy, and membrane dynamics. Despite their small size and modest sequence conservation, proteins like plant LSUs and metazoan SCOCs showcase remarkable functional versatility shaped by evolution, highlighting their potential as adaptive regulators and promising subjects for future biological research.

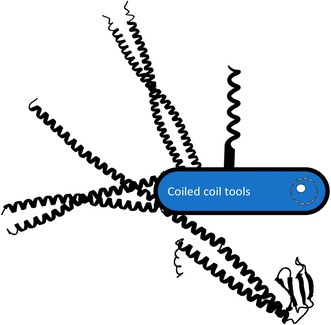

**Abbreviations**
ABA: abscisic acid
ACC: 1‐aminocyclopropane‐1‐carboxylic acid
ACT: modulation of protein activity through direct binding
APS1: ATP sulfurylase 1
ARL1: ADP‐ribosylation factor (ARF)‐like 1
BECN1: Beclin 1
BiFC: bimolecular fluorescence complementation
CAT: catalase
FEZ1: fasciculation and elongation protein zeta 1
FSD2: iron superoxide dismutase
GAPC1: glyceraldehyde‐3‐phosphate dehydrogenase C subunit 1
GRF8: general regulatory factor 8
IP: immunoprecipitation
LSU: upregulated by low sulfur
MiC: colocalization via fluorescence microscopy
mTORC1: mammalian target of rapamycin complex 1
NBR1: next to BRCA1 gene 1
PD: pull‐down
PFDN1‐6: prefoldin 1–6
RAF2: Rubisco assembly factor 2
SCOC: short coiled‐coil
SDIRIP1: SDIR1 interacting protein 1
SiR: sulfite reductase
STING1: stimulator of interferon response cGAMP interactor 1
ULK1: Unc‐51‐like autophagy activating kinase 1
UVRAG: UV irradiation resistance gene
UXT: ubiquitously expressed transcript
Y2H: yeast two‐hybrid

## Introduction

Coiled coils are highly abundant structural domains in the proteomes of all organisms, consisting of two or more alpha‐helical protein structures arranged either in parallel or antiparallel orientation. Their primary sequences typically exhibit a periodic (heptad) arrangement of hydrophobic and polar amino acids, enabling interactions between helices; however, other periodicities also occur. Classical coiled coils can form from two, three, four, five, or more alpha helices, and their architecture can become increasingly complex when multiple coiled coils assemble into higher‐order structures. The physicochemical properties of various coiled‐coil configurations have been extensively reviewed and discussed elsewhere [1, 2]. Multiple computational methods and software tools are available to predict coiled‐coil regions in proteins and facilitate the *de novo* design of specific coiled‐coil architectures [3, 4, 5, 6, 7].

Research on coiled‐coil proteins and peptides intensified following their identification as valuable molecular tools for biomedical material production [8]. Their inherent ability to self‐assemble into highly ordered structures has paved the way for novel applications, including drug and vaccine design, targeted therapeutic delivery, and multivalent epitope display. Additional applications of coiled‐coil peptides include cargo storage, cell culture scaffolding, formation of supramolecular structures such as inclusion bodies (which simplify recombinant protein purification in bacteria), and fibrous assemblies responsive to external stimuli like pH or temperature.

Longer proteins containing one or more coiled‐coil domains are widespread across various organisms [9]. These proteins play crucial roles in multiple cellular processes, including membrane fusion, vesicle tethering, chromosome segregation, signaling, and transcription, and they have been implicated in numerous human diseases [2, 10, 11, 12, 13]. Notably, the coiled‐coil domains in some proteins display evolutionary sequence conservation, while in others, only the structural characteristics and lengths remain conserved [2].

This review focuses specifically on short coiled‐coil proteins, a subset found across plants, multicellular metazoans, and yeasts. The plant‐specific LSU (UPREGULATED BY LOW SULFUR) protein family and the metazoan‐specific SCOC (SHORT COILED‐COIL PROTEINS) family share certain functional similarities despite lacking cross‐kingdom sequence homology. However, they appear to form distinct higher‐order structural arrangements. A third group, prefoldin‐like proteins, possesses a different fold compared to LSUs and SCOCs. The mammalian prefoldin‐like protein UXT (UBIQUITOUSLY EXPRESSED TRANSCRIPT) is the most extensively characterized, although similar proteins are also found in plants.

## The plant LSU family comprises short proteins with a coiled‐coil structure

### General overview of LSU‐like proteins

The plant‐specific family of small coiled‐coil proteins known as LSU has gained attention from several research groups and has been the subject of reviews [14, 15]. This protein family is typically described as ‘plant‐specific’ with ‘unknown function’, since sequence similarity searches identify homologous proteins exclusively within plant species, and their biological roles remain unclear [16]. Plant genomes generally contain multiple genes encoding LSU‐like protein isoforms; for example, *Arabidopsis thaliana* has four *LSU* genes (*LSU1‐4*). LSU‐like proteins are approximately 100 amino acids in length and possess a coiled‐coil domain. Single, multiple, or even quadruple *lsu* knockout mutants remain viable, exhibiting only subtle deviations in growth and developmental patterns compared to wild‐type plants [17]. *LSU* genes were first identified as responsive to sulfur deficiency in Arabidopsis [18, 19, 20] and tobacco [21, 22]. While it is widely assumed that the four LSU isoforms in *A*. *thaliana* may exhibit overlapping functions, differences in their spatio‐temporal expression patterns [14], distinct protein interaction networks, and alterations in the predicted dimer interfaces depending on monomer composition [23] suggest that combinations of LSU monomers into different dimers or multimers could introduce an additional regulatory dimension to their biological activity.

### Protein partners and postulated function

The protein interactomes of LSU proteins from *A. thaliana* have been studied primarily through various global approaches [23, 24, 25, 26]. A limited number of interaction partners have also been identified for the tobacco LSU‐like protein, known as UP9 [21, 27]. However, the overlap among partners discovered in these high‐throughput studies is relatively modest, suggesting that none of these experiments fully captured the complete interactomes. Furthermore, these studies utilized different methodologies, such as yeast two‐hybrid screens employing cDNA libraries or pull‐down assays with tagged LSUs using crude protein extracts from plant tissues. Few LSU partners identified in these high‐throughput analyses have been subsequently verified through additional interaction assays or assessed for the functional impact of LSU proteins on their activities (Table 1).

**Table 1:** Physical interactions of LSU‐like proteins verified by at least two independent methods. All proteins listed are from *Arabidopsis thaliana* unless otherwise noted. ACT, modulation of protein activity through direct binding; BiFC, bimolecular fluorescence complementation; IP, immunoprecipitation; MiC, colocalization via fluorescence microscopy; PD, pull‐down; Y2H, yeast two‐hybrid.

LSU isoform	Interacting partner	Verification methods	References
UP9 (tobacco)	Joka2/NBR1 (tobacco)	Y2H, PD	[28]
UP9 (tobacco)	ACC Oxidase (tobacco)	Y2H, PD, MiC	[27]
LSU1, LSU2, LSU3	NBR1 (At4g24690)	IP, BiFC, Y2H	[23]
LSU1‐4	GRF8 (At5g65430)	IP, BiFC	
LSU1‐4	RAF2/SDIRIP1 (At5g51110)	IP, BiFC	
LSU2, LSU3, ‐LSU4	GAPC1 (At5g65430)	IP, BiFC	
LSU1, LSU2, LSU3	CAT2 (At4g35090)	IP, BiFC, Y2H	
LSU1	CAT1; CAT; CAT3	Y2H, BiFC	[29]
LSU1	SiR (At5g04590)	Y2H, BiFC	[30]
LSU1	APS1 (At3g22890)	IP, BiFC, Y2H	
LSU1, LSU2	FSD2 (At5g51100)	Y2H, BiFC, PD, ACT	[31]
LSU2	AtRAP	Y2H, IP	[32]
LSU2, LSU1	PYL9/RCAR1 (AT1G01360)	Y2H, IP, BiFC	[33]
LSU2, LSU1	NPR4 (AT4G19660)		

The significance of LSU proteins in sulfur assimilation and metabolism was suggested by their strong induction during sulfur starvation [18, 19, 20] and by altered responses to sulfur deficiency or cadmium toxicity observed in quadruple *lsu* mutants and various LSU overexpression lines [34, 35]. Direct interactions between LSU proteins and enzymes involved in sulfate assimilation, such as sulfite reductase (SiR) and ATP sulfurylase 1 (APS1), have been demonstrated [30]. LSU interaction was mapped explicitly to the region containing the active center of SiR, and enhancement of SiR activity by LSU was confirmed *in vitro* [30].

LSU proteins also interact with proteins involved in oxidative stress mitigation, including catalases (CAT1, CAT2, CAT3) and chloroplastic iron superoxide dismutase (FSD2). For catalases, the interaction was localized to their N‐terminal region, suggesting a potential role of LSU in regulating active CAT tetramer formation; however, direct evidence of LSU influencing CAT activity has not yet been provided [29]. In the case of FSD2, a positive impact of LSU on enzyme activity was demonstrated *in vitro* [31].

The interactions between LSU and NBR1 (Next to BRCA1 gene 1) proteins have been documented in both tobacco and *A. thaliana*. NBR1 is a selective autophagy receptor responsible for recognizing and delivering cargo targeted for autophagic degradation. Autophagy is an evolutionarily conserved catabolic process that degrades cellular macromolecules and organelles through encapsulation in double‐membraned vesicles, followed by delivery to lysosomes or vacuoles. Selective autophagy receptors specifically recognize and facilitate the degradation of cargo marked for disposal (reviewed in [36]). NBR1 is among plants' best‐characterized selective cargo receptors, representing a hybrid of two mammalian proteins, p62/sequestosome 1 (SQSTM1) and NBR1. The functional significance of LSU‐NBR1 interactions remains unclear.

Additional confirmed interaction partners of LSU proteins, verified through multiple independent methods, include 1‐aminocyclopropane‐1‐carboxylic acid (ACC) oxidase, involved in ethylene synthesis; general regulatory factor 8 (GRF8), a member of the 14–3‐3 family known to interact with the BZR1 transcription factor involved in brassinosteroid signaling; Rubisco assembly factor 2 (RAF2), also known as SDIR1 interacting protein 1 (SDIRIP1), implicated in abscisic acid (ABA) signaling; glyceraldehyde‐3‐phosphate dehydrogenase C subunit 1 (GAPC1); ABA receptor PYL9; salicylic acid receptor NPR4; and RNA‐binding protein AtRAP. Relevant references are summarized in Table 1.

In summary, LSU‐like proteins participate in numerous protein–protein interactions, yet analysis of their partners provides limited clues regarding their precise biological functions or roles in specific molecular processes. Several generalized functions have been tentatively attributed to LSU proteins. They are proposed as hubs that integrate plant responses to diverse environmental stresses [37] and potentially facilitate intercellular trafficking of various proteins [23]. Recent work have further suggested their involvement in plant stress responses through modulation of phytohormone signaling pathways, including those regulated by abscisic acid, ethylene, auxin, jasmonic acid, and salicylic acid [14]. Consequently, LSU functions are likely extending beyond their initially hypothesized roles in sulfur starvation responses. LSU proteins may function as molecular scaffolds, assembling protein complexes in response to stress, influencing metabolic pathways such as sulfate assimilation, and coordinating cellular redox states during nutrient limitation.

## SCOC‐like proteins

### Human SCOC are involved in vesicular trafficking and autophagy

Initially identified as specific binding partners of ADP‐ribosylation factor (ARF)‐like 1 (ARL1), a GTPase involved in membrane trafficking, SCOC proteins colocalize with Golgi membranes. This interaction is quickly reversed by brefeldin A, a potent inhibitor of the secretory system and vesicle formation [38]. The ARF protein family regulates vesicle biogenesis and trafficking, but also influences lipid metabolism, amino acid sensing via mTORC1 (mammalian target of rapamycin complex 1), cytoskeletal dynamics, ciliogenesis, and lysosomal and mitochondrial morphology and function [39].

Human SCOC was identified as essential for starvation‐induced autophagy through a genome‐wide siRNA screen in HEK293 cells, interacting with FEZ1 (fasciculation and elongation protein zeta 1) and UVRAG (UV irradiation resistance gene) [40, 41]. SCOC interactions with FEZ1 and UVRAG regulate the activities of both the ULK1/Atg1 kinase complex and the UVRAG‐BECN1‐Vps34 complex, essential for phagophore initiation and autophagosome formation/maturation, respectively. SCOC binding to FEZ1 is unaffected by starvation, whereas interaction with UVRAG decreases upon starvation, releasing ULK1 and UVRAG to activate and initiate autophagy. Thus, SCOC mediates crosstalk between key complexes regulating autophagy at multiple steps [40, 41].

A complex comprising FEZ1, SCOC, ULK1, and NBR1, a selective autophagy receptor, has been reported in mice and humans [42, 43], potentially influencing neural development and neuronal function restoration via autophagy modulation. However, molecular details remain unexplored, including direct SCOC‐NBR1 interactions.

Further emphasizing SCOC's role in autophagy, interactions between specific SCOC isoforms and human ATG8 proteins were recently described [44]. ATG8 proteins, embedded in autophagosomal membranes via phosphatidylethanolamine conjugation, interact with cargo receptors (e.g., p62, NBR1) and adaptors during selective autophagy. ATG8 interactions generally occur via a hydrophobic patch with two pockets (W and L), known as the LIR/AIM docking site (LDS), or an alternative ubiquitin‐interactive motif‐docking site (UDS). The ATG8‐interacting motif (AIM or LIR) was identified in the N‐terminal region of some human SCOC isoforms [44]. SCOC isoforms, varying from 54 to 122 residues with distinct N‐terminal domains but identical C‐terminal regions, bind several ATG8 homologs (GABARAP, GABARAPL1, LC3A, LC3C) via the LIR motif. SCOC‐ATG8 interactions are enhanced by phosphorylation near the LIR motif, with direct phosphorylation demonstrated by ULK(1–3) and TBK1 kinases *in vitro* [44]. These findings support SCOC's proposed regulatory role in mammalian autophagy, corroborating earlier data indicating SCOC colocalization with ATG9 and LC3 [41] beyond its previously described association with the Golgi [38].

### The concept of ‘dual adapter’ formed by the SCOC‐FEZ1 complex

The FEZ family of proteins participates in several biological processes, including neuronal development, neurological disorders, viral infections, and autophagy. FEZ proteins function both in the cytoplasm and the nucleus, acting as versatile adapters in kinesin‐mediated transport [45]. FEZ1 proteins form dimers via their N‐terminal regions, stabilized by disulfide bonds, whereas the C‐terminal region, containing the coiled‐coil domain, remains available for additional protein interactions. Two studies conducted in 2013 investigated the structure of the SCOC‐FEZ1 complex and the involvement of the FEZ1 C‐terminal region in complex formation [46, 47]. Interestingly, these studies reached slightly different conclusions: One study suggested that the FEZ1‐SCOC complex is tetrameric, comprising a FEZ1 homodimer bound to two SCOC molecules [46]. In contrast, the other study concluded that SCOC and FEZ1cc (a FEZ1 fragment containing its coiled‐coil region) form a stable, homogeneous complex with a molecular weight of approximately 120 kDa. Such a value would correspond to a complex containing six copies of each protein, assuming a 1 : 1 stoichiometry, and indicate that SCOC dimerization is essential for complex formation [47]. Despite these differences, the interaction between SCOC and the FEZ1 C‐terminal region, forming a protein–protein docking domain, likely modulates FEZ1's scaffolding functions. In the nucleus, this interaction could alter FEZ1's role as a transcriptional regulator, while in the cytoplasm, it may facilitate proper cargo loading onto kinesin machinery [45].

### SCOC‐like proteins in other Metazoa

SCOC proteins, also referred to as SCOCO, are ubiquitously present in various animal tissues [48]. Beyond humans, SCOC proteins have also been identified in other metazoan species (Table 2).

**Table 2:** Selected database entries for SCOCs from different *Metazoa*.

Organism/protein name	Locus	UniProt entry
*Homo sapiens* SCOC isoforms 1–6	NP_001146956.2 NP_001147135.1 NP_001147024.2 NP_115936.2 NP_001147107.1 NP_001147162.1	Q9UIL1‐1 (159aa) Q9UIL1‐2 (122aa) Q9UIL1‐3 (121 aa) Q9UIL1‐4 (134 aa) A0A804E055 (54 aa) A0A0C4DGB0 (82 aa)
*Drosophila melanogaster* SCOCO‐like	Dmel\CG5934 GEO08385p1	Q9VB51_DROME (135 aa)
*Caenorhabditis elegans* SCOCO‐like/ UNC‐69	NP_001122717.1 NP_001022753.1 NP_001022752.1	G5EDQ5 (122 aa) A7LPF8 (176 aa) Q9U377 (108 aa)
*Saccharomyces cerevisiae* SCOCO‐like/ SLO1	YER180C‐A	Q3E784 (85 aa)

In *Caenorhabditis elegans*, the SCOC orthologue UNC‐69 (108 amino acids) is ubiquitously expressed throughout the nervous system and in non‐neuronal cells across all developmental stages. UNC‐69 interacts with UNC‐76, the FEZ1 orthologue, and their complex regulates axonal growth by controlling vesicular transport along axons [48]. Mutants lacking functional UNC‐69 exhibit defects in axonal growth. Additionally, UNC‐76 interacts with UNC‐51 (ATG1/ULK1), and both proteins colocalize in distinct, perinuclear dots within the soma, a localization dependent on UNC‐116 (kinesin heavy chain, KHC). UNC‐76 (FEZ1) utilizes distinct interaction regions for UNC‐51 (ULK1) and UNC‐69 (SCOC) [48]. The authors conclude that UNC‐69 and UNC‐76 form a conserved protein complex essential for regulating vesicular transport along axons in *C. elegans*, although dendritic development and protein transport into dendrites remain unaffected in unc‐69 mutants. This complex may also participate in synapse formation. Interestingly, in *Drosophila melanogaster*, a FEZ1 homolog binds the tail domain of the kinesin heavy chain (KHC), suggesting a conserved role in kinesin‐dependent axonal transport [49].

A recent preprint further reports that in *C. elegans*, UNC‐69 and UNC‐76 recruit spectrin to kinesin‐1, supporting its slow transport and distribution along axons. Notably, the UNC‐69 function can be circumvented by directly linking spectrin to kinesin [50]. Spectrin is a cytoskeletal protein associated with the intracellular side of the plasma membrane in eukaryotic cells, critical for maintaining membrane integrity and cytoskeletal organization [51, 52]. Spectrin proteins appear to be specific to animal evolution [53]; however, proteins containing spectrin repeats have also been identified in plants [54]. One example is MAP65‐1 in *A. thaliana*, a protein containing four spectrin repeats involved in microtubule reorganization [55]. Interestingly, the microtubule‐associated protein MAP65‐1a was previously identified as a partner of UP9, an LSU‐like protein from tobacco [15, 27].

In *Saccharomyces cerevisiae*, the SCOC‐like protein SLO1 has been identified. Unlike mammalian SCOC, SLO1 does not localize to the Golgi apparatus [56]. Nevertheless, similar to human SCOC, SLO1 interacts with one of the yeast ADP‐ribosylation factor‐like (ARF‐like) proteins, Arl3p. Deletion of SLO1 does not affect yeast cell viability [56].

## Specific and common features of plant LSU and human SCOC proteins

### Protein sequence and structure

**Fig. 1:**
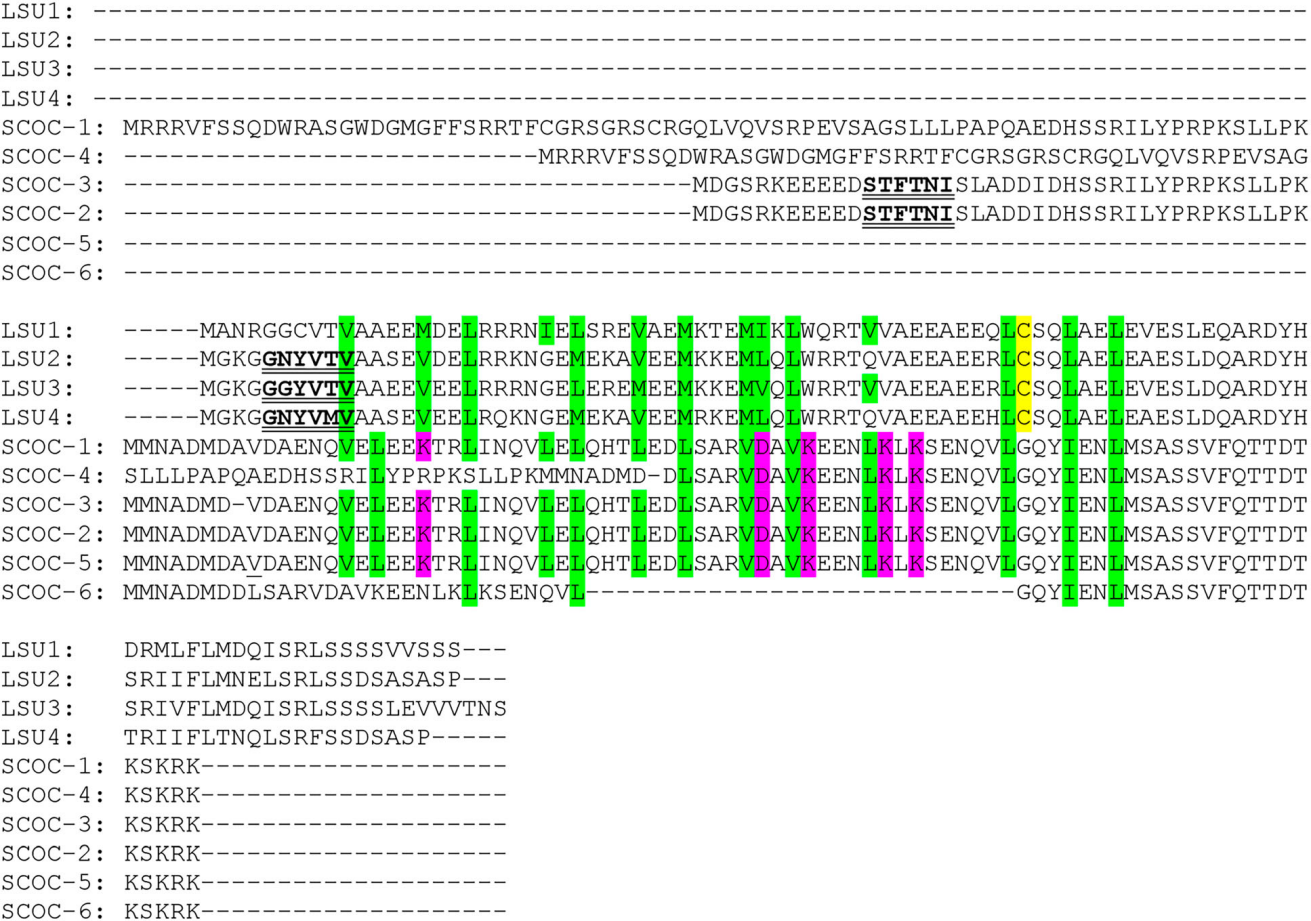
Amino acid sequences alignment of *Arabidopsis thaliana* LSU isoforms: LSU1 (At3g49580), LSU2 (At5g24660), LSU3 (At3g49570), and LSU4 (At5g24655) and *Homo sapiens* SCOC isoforms: SCOC‐1 (Q9UIL1‐1), SCOC‐4 (Q9UIL1‐4), SCOC‐3 (Q9UIL1‐3), SCOC‐2 (Q9UIL1‐2), SCOC‐5 (A0A0C4DGB0), and SCOC‐6 (A0A804E055) using the muscle software (https://www.ebi.ac.uk/jdispatcher/msa) followed by a slight manual alignement of the residues involved in coiled‐coil formation (highlighted green). Purple indicates residues in SCOC potentially responsible for structural differences between SCOC and LSU; yellow indicates conserved cysteine residues in all plant LSU‐like proteins. Putative LIR/AIM motifs identified by ‘iLIR – *in silico* identification of functional LC3 Interacting Region Motifs’ (https://ilir.warwick.ac.uk/) are underlined.

The LSU‐like proteins from *A*. *thaliana* and SCOC proteins from *Homo sapiens* do not share any conserved regions or domains. They are classified by distinct InterPro domains (https://www.ebi.ac.uk/interpro/): IPR039282 (LSU‐like proteins) and IPR019357 (SCOC proteins). These proteins exhibit no significant sequence similarity except for a conserved spatial arrangement of residues predicted to participate in coiled‐coil formation (Fig. 1).

Notably, a cysteine residue conserved in all plant LSU‐like proteins, predicted by structural modeling to be externally exposed in the dimer, is absent in SCOC proteins. This cysteine residue may play an essential role in redox regulation, as cysteines can undergo numerous posttranslational modifications, including S‐nitrosylation (SNO), S‐glutathionylation (SSG), S‐acylation, S‐cysteinylation, S‐sulfhydration, S‐sulfenylation (Cys‐SOH), and intra‐ or intermolecular disulfide bonds [57]. The presence of this residue could thus reflect a functional distinction between plant LSU proteins and animal SCOC proteins, particularly in stress signaling and protection of antioxidant enzymes (e.g., CAT, FSD2).

**Fig. 2:**
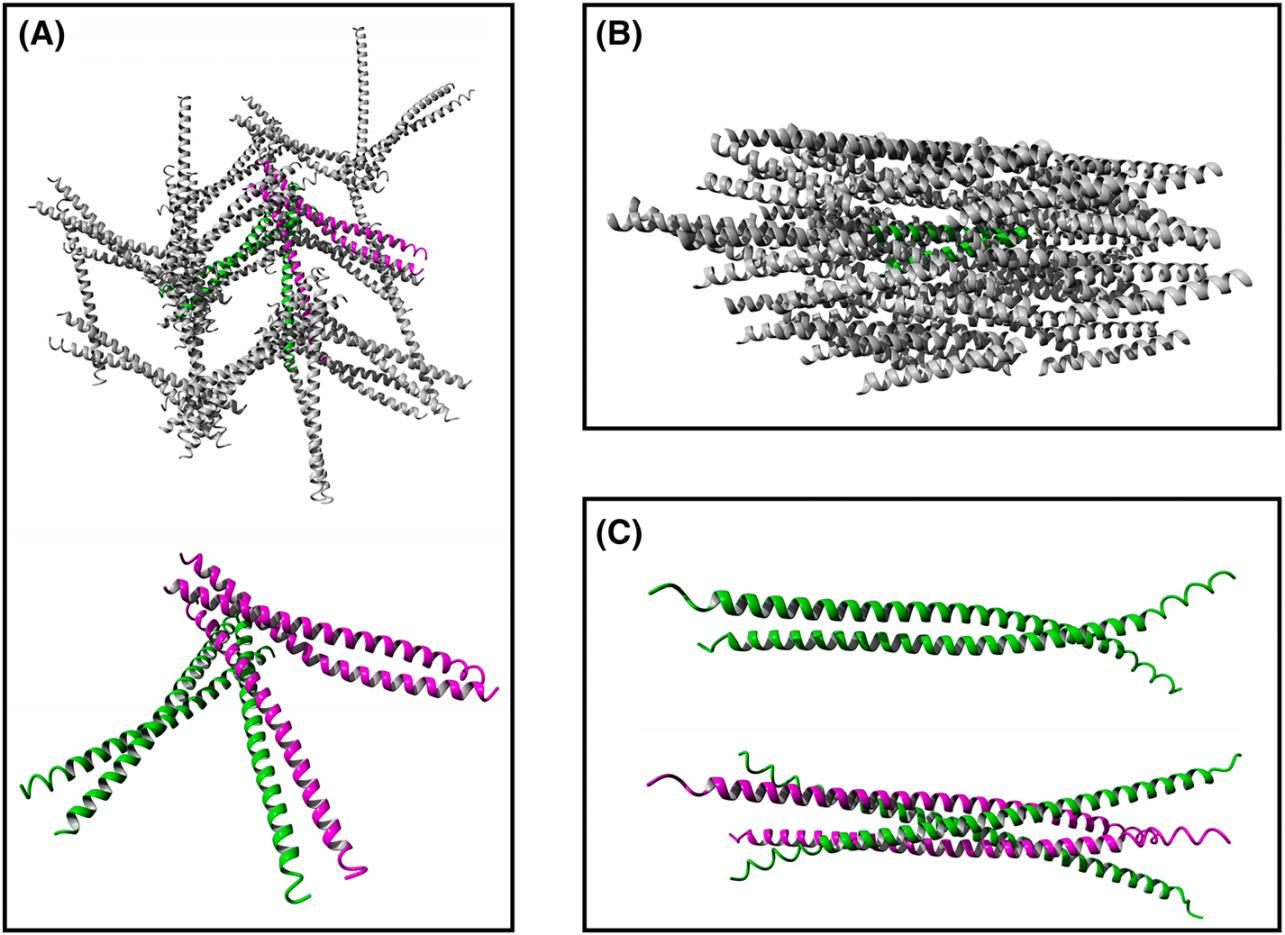
Comparison of the 3D packing arrangements of short coiled‐coil proteins. (A) SCOC molecules (PDB: 4BWD) form a diffuse network of hexameric units in the crystal structure (upper image), where each unit cell (green or magenta) contains three SCOC molecules, and these six SCOC molecules collectively form a loose trimer of coil‐coiled dimers (lower image). (B) Crystal packing of antiparallel coiled‐coil dimers (apCC‐Di; PDB: 7Q1R), illustrating a typical example of higher‐order structures observed in coiled‐coil protein crystals. (C) The alphafold‐derived model of SCOC homotetramers displays an antiparallel four‐helix coil‐coiled arrangement (lower image), which is incompatible with the parallel coiled‐coil SCOC dimer model (upper image). All the structures were visualized using the Yasara Structure package.

Structural analyses suggest that although both SCOC and LSU proteins form parallel coiled‐coil homodimers, subsequent organization differs significantly. The crystal structure of SCOC (PDB: 4BWD) reveals an atypical asymmetric parallel coiled‐coil dimer with one helix significantly bent. Intriguingly, the crystal unit cell contains three molecules, two forming an asymmetric parallel dimer and the third forming a symmetric parallel dimer with a molecule from an adjacent crystal cell. Examination of crystal packing (PDB: 4BWD) reveals an unusual diffuse network arrangement rather than the typical ordered, higher‐order coiled‐coil assemblies (Fig. 2A,B). LSU structures are not yet crystallographically determined, but AlphaFold models suggest that LSU proteins form higher‐order oligomers, specifically antiparallel dimers of parallel dimers (homotetramers) [29], potentially leading to fibril‐like structures common in other coiled‐coil proteins (Fig. 2B). In contrast, AlphaFold models of SCOC homotetramers suggest antiparallel four‐stranded coiled coils without parallel dimer structures (Fig. 2C).

These structural differences imply that SCOC coiled‐coil dimers may be inherently less stable compared to LSU dimers. Experimental evidence supports this interpretation: LSU proteins bind catalase in a stable coiled‐coil dimeric form [29], whereas the SCOC‐FEZ1 complex exhibits a 2 : 2 stoichiometry, forming a symmetric dimer of 1 : 1 heterodimers (FEZ1 : SCOC) [46]. Additionally, SAXS analysis indicates that SCOC proteins can exist as monomers in solution [46]. Conversely, all four LSU family proteins preferentially form stable parallel coiled‐coil dimers, with some combinations (LSU1‐LSU3 and LSU2‐LSU4) capable of forming heterodimers [23], while alphafold‐modeled LSU1 tetramers consist of two parallel coiled‐coil dimers [29].

### Protein partners and function

**Fig. 3:**
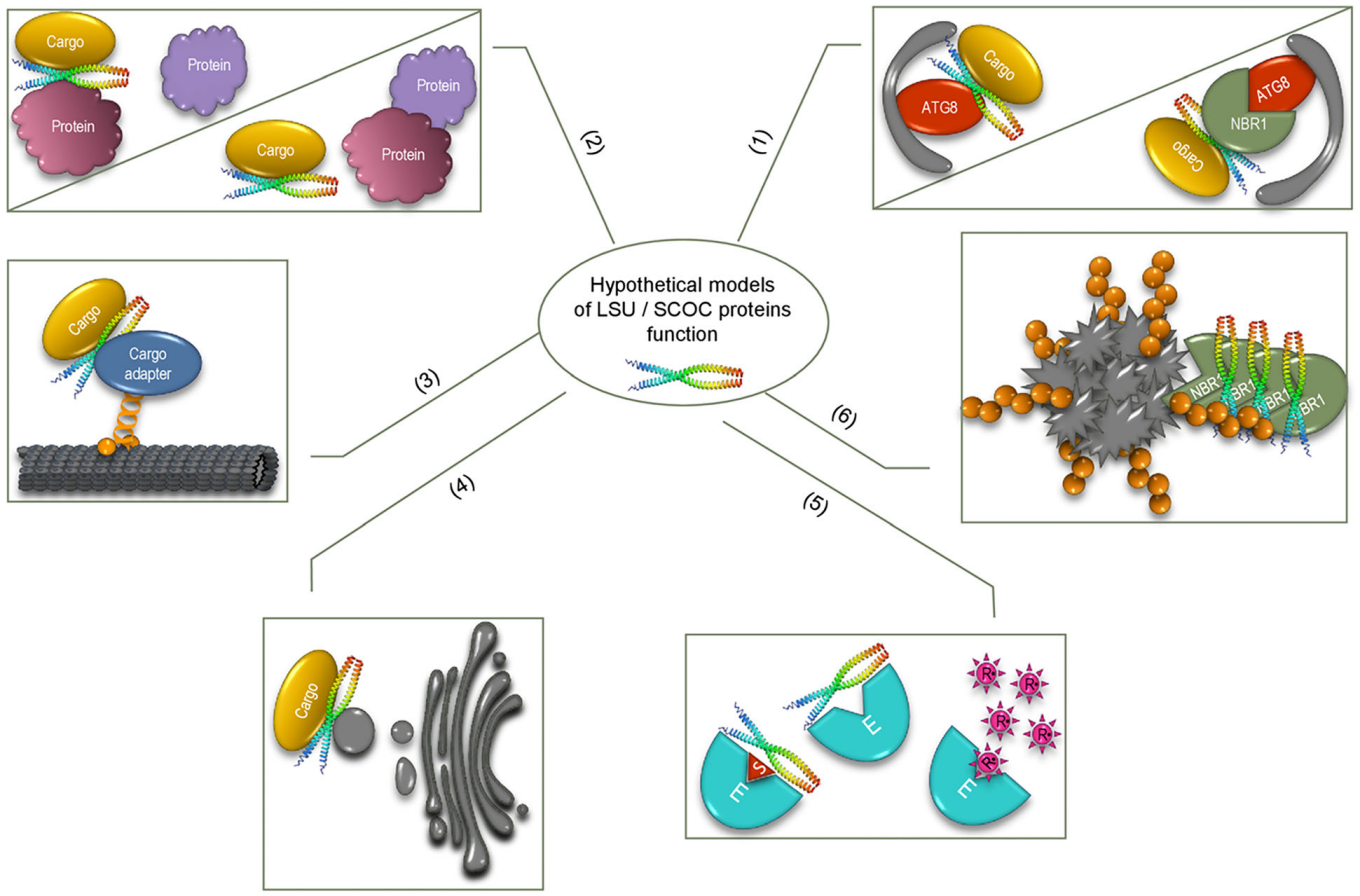
Hypothetical models illustrating potential cellular functions of LSU and SCOC proteins. (1) Acting as autophagy adapters independently or in complex with NBR1 or other selective autophagy receptors; (2) Facilitating or inhibiting interactions between cargo proteins and other cellular components; (3) Serving as cargo‐motor adapters or co‐adapters for microtubule‐based transport; (4) Promoting cargo association with transport vesicles and/or vesicular trafficking systems; (5) Stabilizing enzyme active sites and protecting proteins against oxidative stress; (6) Modulating selective autophagy by enhancing aggregation of selective autophagy receptors.

A common feature of LSU and SCOC proteins is their involvement in numerous protein–protein interactions, most identified through high‐throughput methods with few examined in detail. Gene ontology (GO) term enrichment analysis has not clearly revealed shared biological processes, molecular functions, or protein categories within their respective interactomes. Interestingly, both protein families associate with autophagy: LSU proteins interact with NBR1, while SCOC proteins facilitate starvation‐induced autophagy in human cells and bind ATG8 proteins. However, many interaction partners of both protein families show no direct relation to autophagy. Thus, we propose a hypothetical model where LSU and SCOC proteins modulate their cargo proteins' interaction capacities, regulate their stability and activity, influence their intracellular movement, or target them for degradation via autophagy (Fig. 3).

## Another type of short coiled‐coil proteins—prefoldin‐like

Some members of the prefoldin family also fulfill the criteria for short proteins containing coiled‐coil domains. Prefoldin is a well‐characterized cytoplasmic co‐chaperone complex comprising six subunits—two alpha (PFDN3 and PFDN5) and four beta (PFDN1, PFDN2, PFDN4, and PFDN6)—and is evolutionarily conserved in higher eukaryotes, including humans and plants. Prefoldins assist in folding nascent polypeptides, accelerate the maturation of cytoskeletal proteins (actin and tubulin), participate in transcription elongation, maintain protein homeostasis during cellular stress, and have numerous other, not yet fully understood non‐canonical functions, such as chromatin remodeling, transcript splicing, protein quality control and aggregation, and viral infection regulation (reviewed by [58]). Interactions between prefoldins and their substrates rely on coiled‐coil motifs, with individual prefoldin subunits interacting with distinct proteins and potentially having different functional roles.

The prefoldin complex is formed by its subunits, but there are also prefoldin‐like proteins that do not clearly form part of this complex (reviewed by [59]). One example is UXT (ubiquitously expressed prefoldin‐like chaperone), widely expressed across various human and mouse tissues. UXT is a small protein consisting of 157 amino acids and contains two helical regions separated by a non‐helical segment composed of beta‐strands and coils. These two helical regions are predicted to form an antiparallel coiled‐coil structure, with a loop region creating a hinge (AlphaFoldDB: Q9UBK9). Classified as a prefoldin alpha‐like subunit, UXT is thought to form oligomers. UXT interacts with numerous proteins and has diverse functions; for instance, it is an important centrosomal component, binds gamma‐tubulin, and is essential for cell viability [60]. UXT modulates autophagy and autophagic degradation of various proteins, interacts directly with mTOR to enhance its activity [61], and facilitates STING1 (stimulator of interferon response cGAMP interactor 1) interaction with p62, attenuating the CGAS‐STING1 signaling pathway critical for innate immune responses [62]. Additionally, UXT positively regulates p62‐dependent aggrephagy in mammals [63]. Thus, similarly to LSU and SCOC, UXT seems involved in autophagy.

Prefoldins are evolutionarily conserved and also present in plants [64, 65]. The *Arabidopsis thaliana* genome contains six PFDN genes and at least two genes encoding prefoldin‐like proteins structurally similar to UXT (At1g49245 and At1g26660). However, plant prefoldin‐like proteins remain poorly characterized.

## Closing remarks, limitations of current studies, and future perspectives

Plant and animal cells share both similarities and differences. Many cellular functions, such as autophagy, vesicular transport, and cytoskeletal organization, are evolutionarily conserved. Conversely, chloroplasts and cell walls in plants impose constraints, resulting in differences in cytoskeletal architecture, motor proteins, morphology, and cell division. LSU proteins predominantly appear involved in adapting to environmental stress, whereas SCOC proteins mainly regulate homeostatic cellular processes. Until now, SCOC‐like proteins have been considered animal‐specific, and LSU‐like proteins plant‐specific, respectively. These proteins exhibit no sequence similarity apart from a similar (though not identical) arrangement of residues involved in coiled‐coil formation. Nevertheless, despite the lack of sequence conservation, they share structural and functional similarities to some extent, particularly roles related to cellular trafficking and autophagy. These proteins function as facilitators rather than essential components, acting as versatile regulators within metabolic networks.

Research on short coiled‐coil proteins is challenging due to their pleiotropic functions and numerous interaction partners involved in diverse metabolic pathways. Progress in understanding their cellular roles may be advanced by (a) comprehensive, high‐throughput analysis of their interactomes and phenotyping knockout mutants under varying conditions and developmental stages and (b) detailed investigations of their effects on specific protein partners and processes.

Several unresolved questions remain regarding these proteins in both plant and animal cells: (a) Which processes are influenced or unaffected by these proteins? Are they essential under specific conditions? (b) What mechanisms underlie their actions, and what structural details characterize their interactions with diverse targets? More structural data are needed. (c) Organisms typically contain multiple isoforms of these proteins. Are these isoforms functionally specialized, possibly due to variations in binding efficiencies with specific targets? Are heteromultimer formations functionally distinct? (d) Are structural and functional similarities between animal and plant proteins the result of convergent evolution, or does their sequence divergence reflect the divergent evolution of ancestral genes? (e) Can insights from one biological kingdom be extrapolated to understand their roles in the other?

## Conflict of interest

The authors declare no conflict of interest.

## Author contributions

AS designed the outline and drafted the manuscript. MS generated Figs 1 and 3 and graphical abstract. JP generated Fig. 2. All authors edited and revised the manuscript and the graphics, reviewed and approved the final version of the manuscript.
